# Differential and shared effects of eicosapentaenoic acid and docosahexaenoic acid on serum metabolome in subjects with chronic inflammation

**DOI:** 10.1038/s41598-021-95590-7

**Published:** 2021-08-11

**Authors:** Wan-Chi Chang, Jisun So, Stefania Lamon-Fava

**Affiliations:** 1 Cardiovascular Nutrition Laboratory, Jean Mayer USDA Human Nutrition Research Center On Aging, Tufts University, 711 Washington Street, Boston, MA 02111 USA; 2 Gerard J. and Dorothy R. Friedman School of Nutrition Science and Policy, Tufts University, Boston, USA

**Keywords:** Cell biology, Diseases, Biochemistry, Lipids, Metabolomics

## Abstract

The omega-3 fatty acids eicosapentaenoic acid (EPA) and docosahexaenoic acid (DHA) affect cell function and metabolism, but the differential effects of EPA and DHA are not known. In a randomized, controlled, double-blind, crossover study, we assessed the effects of 10-week supplementation with EPA-only and DHA-only (3 g/d), relative to a 4-week lead-in phase of high oleic acid sunflower oil (3 g/day, defined as baseline), on fasting serum metabolites in 21 subjects (9 men and 12 post-menopausal women) with chronic inflammation and some characteristics of metabolic syndrome. Relative to baseline, EPA significantly lowered the tricarboxylic acid (TCA) cycle intermediates fumarate and α-ketoglutarate and increased glucuronate, UDP-glucuronate, and non-esterified DHA. DHA significantly lowered the TCA cycle intermediates pyruvate, citrate, isocitrate, fumarate, α-ketoglutarate, and malate, and increased succinate and glucuronate. Pathway analysis showed that both EPA and DHA significantly affected the TCA cycle, the interconversion of pentose and glucuronate, and alanine, and aspartate and glutamate pathways (FDR < 0.05) and that DHA had a significantly greater effect on the TCA cycle than EPA. Our results indicate that EPA and DHA exhibit both common and differential effects on cell metabolism in subjects with chronic inflammation and some key aspects of metabolic syndrome.

## Introduction

Chronic inflammation is a hallmark of several common cardiometabolic diseases such as metabolic syndrome, obesity, type 2 diabetes mellitus and cardiovascular disease (CVD)^1^. Recent advances in metabolomics tools and analysis have increased our ability to uncover metabolic pathways affected by these diseases and by treatments that ameliorate them^2,3^. Metabolic syndrome, which is highly prevalent in obesity and predisposes to both diabetes and CVD, has been associated with plasma levels of various metabolites: biogenic amines such as choline, L-carnitine and trimethylamine- N-oxide (TMAO), amino acids such as valine, alanine, glutamate and tyrosine, glucose, and the intermediates of energy metabolism such as pyruvate^4,5^. In animal models of diet-induced obesity and CVD, the long chain omega-3 fatty acids (n-3 FA) eicosapentaenoic acid (EPA) and docosahexaenoic acid (DHA) have been shown to reduce inflammation and improve insulin sensitivity and glucose metabolism^6^. However, in human prospective and randomized intervention studies the evidence of these beneficial effects of n-3 FA supplementation on glucose homeostasis is mixed^6,7^. Nevertheless, reduction in CVD risk by n-3 FA, more by EPA than DHA, has been reported^8,9^, Within cells, n-3 FA are not only important components of the cell membrane, but also have important roles in a number of metabolic processes by regulating the activity of enzymes, acting as signaling molecules, and serving as ligands for transcription factors^10^. A direct comparison between EPA and DHA regarding their effects on metabolism in humans has not been performed.

The objective of this study was to gain a better understanding of the common and differential effects of EPA and DHA on the serum metabolome and associated metabolic pathways in individuals with chronic inflammation and multiple features of metabolic syndrome.

## Results

### Subjects’ characteristics

The mean age (± SD) of subjects who completed the study (n = 21) was 61 ± 6 years (Table 1). All subjects were overweight or obese, with a mean body mass index (BMI) of 32.2 kg/m^2^ and mean waist circumference above the sex-specific values for abdominal obesity. Subjects had borderline high fasting plasma blood glucose and triglyceride (TG) concentrations.

**Table 1:** Characteristics of subjects at screening visit.

	All (*n* = 21)	Male (*n* = 9)	Female (*n* = 12)
Age (y)	61 ± 6	59 ± 5	64 ± 6
Weight (kg)	92.7 ± 20.4	102.5 ± 17.0	85.3 ± 20.1
BMI (kg/m^2^)	32.2 ± 6.6	32.2 ± 5.6	32.2 ± 7.5
Waist circumference (cm)	104 ± 15	111 ± 11	100 ± 15
SBP (mmHg)	130 ± 16	132 ± 10	128 ± 19
DBP (mmHg)	79 ± 12	87 ± 8	73 ± 11*
Fasting glucose (mg/dL)	100 ± 10	101 ± 11	99 ± 10
Fasting TG (mg/dL)	144 ± 46	129 ± 31	155 ± 53

Values are reported as mean ± SD.

*P < 0.05, women versus men.

BMI, body mass index; SBP, systolic blood pressure; DBP, diastolic blood pressure; TG, triglyceride.

Screening values in men and women were similar, except for diastolic blood pressure, which was significantly lower in women than men (*P* = 0.003).

### Effects of EPA and DHA on biochemical parameters

We have previously reported the effect of EPA and DHA supplementation on the plasma phospholipid content of arachidonic acid (AA), EPA and DHA^11^. The median molar percent (mol%) of AA at baseline and at the end of the EPA and DHA phases was 11.5, 10.5 and 9.5, respectively. The median mol% of EPA was 0.7, 5.3 and 1.5, respectively, and of DHA was 2.8, 3.1 and 7.7, respectively.

Biochemical parameters at baseline and after EPA and DHA supplementation are shown in Table 2. Relative to baseline, EPA supplementation significantly increased serum concentrations of lactate dehydrogenase whereas DHA supplementation significantly increased serum albumin concentrations. There was no significant difference between the EPA- and DHA-induced changes.

**Table 2:** Serum concentrations of biochemical parameters at baseline and after EPA and DHA supplementation^1^.

	Baseline	EPA	∆EPA from baseline^2^	DHA	∆DHA from baseline^2^	FDR-∆EPA vs. ∆DHA^3^
Albumin (g/dL)	4.03 ± 0.20	4.11 ± 0.18	0.08 ± 0.20	4.16 ± 0.22	0.12 ± 0.17*	0.36
Total protein (g/dL)	6.60 ± 0.30	6.71 ± 0.33	0.10 ± 0.31	6.78 ± 0.38	0.17 ± 0.31	0.36
SGPT (mU/mL)	6.48 ± 2.99	7.87 ± 3.20	1.33 ± 3.94	8.10 ± 3.95	1.62 ± 3.53	0.64
SGOT (mU/mL)	12.38 ± 3.34	13.24 ± 3.97	0.86 ± 3.18	13.57 ± 3.38	1.19 ± 2.56	0.82
LDH (U/L)	110.62 ± 21.37	120.38 ± 22.28	9.76 ± 17.18*	118.33 ± 21.44	7.71 ± 15.14	0.35
ALP (U/L)	58.90 ± 16.62	61.71 ± 18.52	2.81 ± 10.91	57.76 ± 17.60	− 1.14 ± 10.10	0.07
BUN (mg/dL)	16.43 ± 4.23	15.43 ± 3.68	− 1.00 ± 2.57	16.38 ± 5.17	− 0.05 ± 3.17	0.46
Creatinine (mg/dL)^4^	0.87 ± 0.19	0.89 ± 0.19	0.01 ± 0.08	0.89 ± 0.19	0.02 ± 0.19	0.79
Glucose (mg/dL)	98.43 ± 8.07	100.29 ± 7.29	1.86 ± 5.75	98.95 ± 9.19	0.52 ± 6.10	0.35
NEFA (mmol/L)	0.18 ± 0.09	0.21 ± 0.13	0.03 ± 0.09	0.20 ± 0.13	0.02 ± 0.11	0.70
Uric acid (mg/dL)^4^	5.86 ± 1.34	5.79 ± 1.41	− 0.07 ± 0.76	5.79 ± 1.30	− 0.07 ± 0.83	0.77
Total bilirubin (mg/dL)	0.56 ± 0.17	0.59 ± 0.19	0.03 ± 0.17	0.58 ± 0.16	0.02 ± 0.19	0.88

^1^Values are reported as unadjusted mean ± SD. To correct for skewed distribution, log-transformation was applied before analysis.

^2^Comparison of changes from baseline to EPA or DHA was assessed using a liner mixed-effects model.FDR-adjusted* P* values; *FDR < 0.05.

^3^Pairwise comparison between EPA and DHA (changes form baseline) were conducted using the *lsmeansLT* function from the *lmerTest* package for the linear mixed effect model.

^4^Creatinine (*p* = 0.0231) and uric acid (*p* = 0.04) showed a significant sequence effect; therefore, only data from the first period was used to run ANCOVA analysis, with baseline value as a covariate.

SGPT, serum glutamic-pyruvic transaminase; SGOT, serum glutamic-oxaloacetic transaminase; LDH, lactate dehydrogenase; ALP, alkaline phosphatase; BUN, blood urea nitrogen; NEFA, non-esterified fatty acids.

### Effects of EPA and DHA on serum primary metabolites

**Figure 1:**
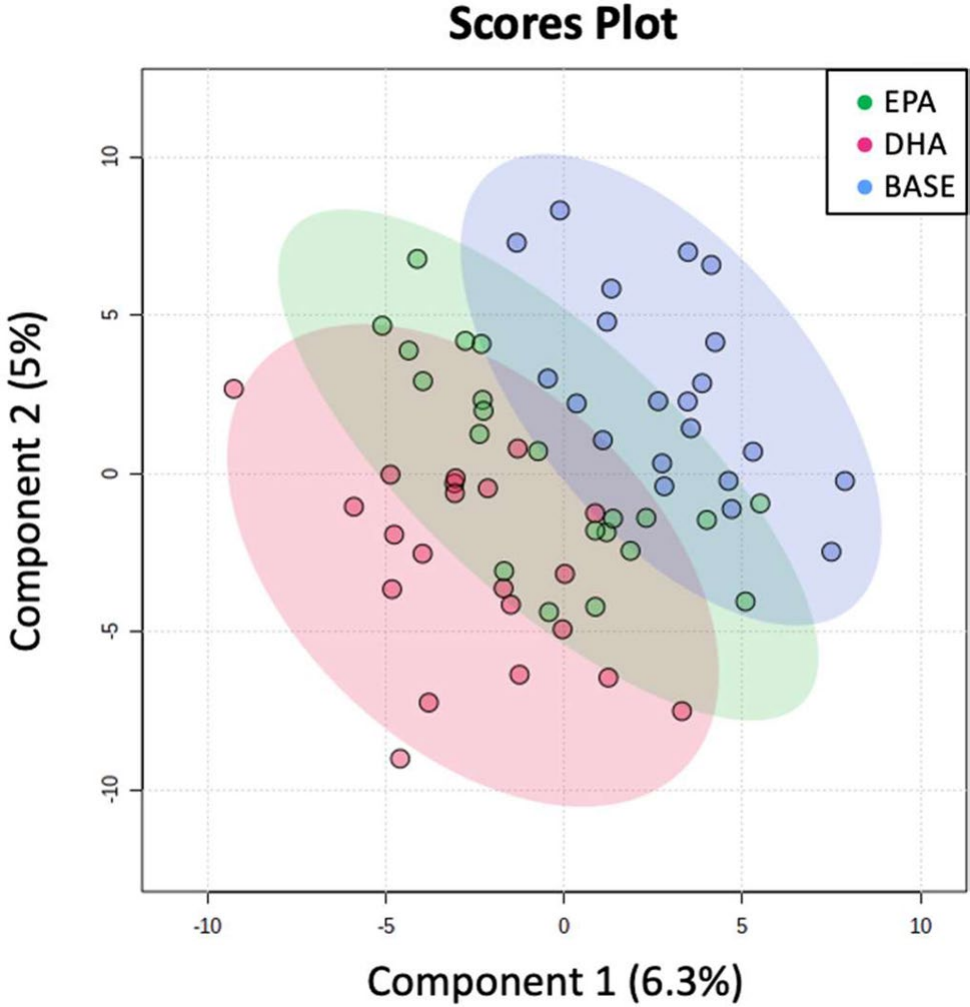
PLS-DA score plot. Primary metabolites are shown at baseline (blue) and at the end of the EPA (green) and DHA (red) supplementation phases.

**Figure 2:**
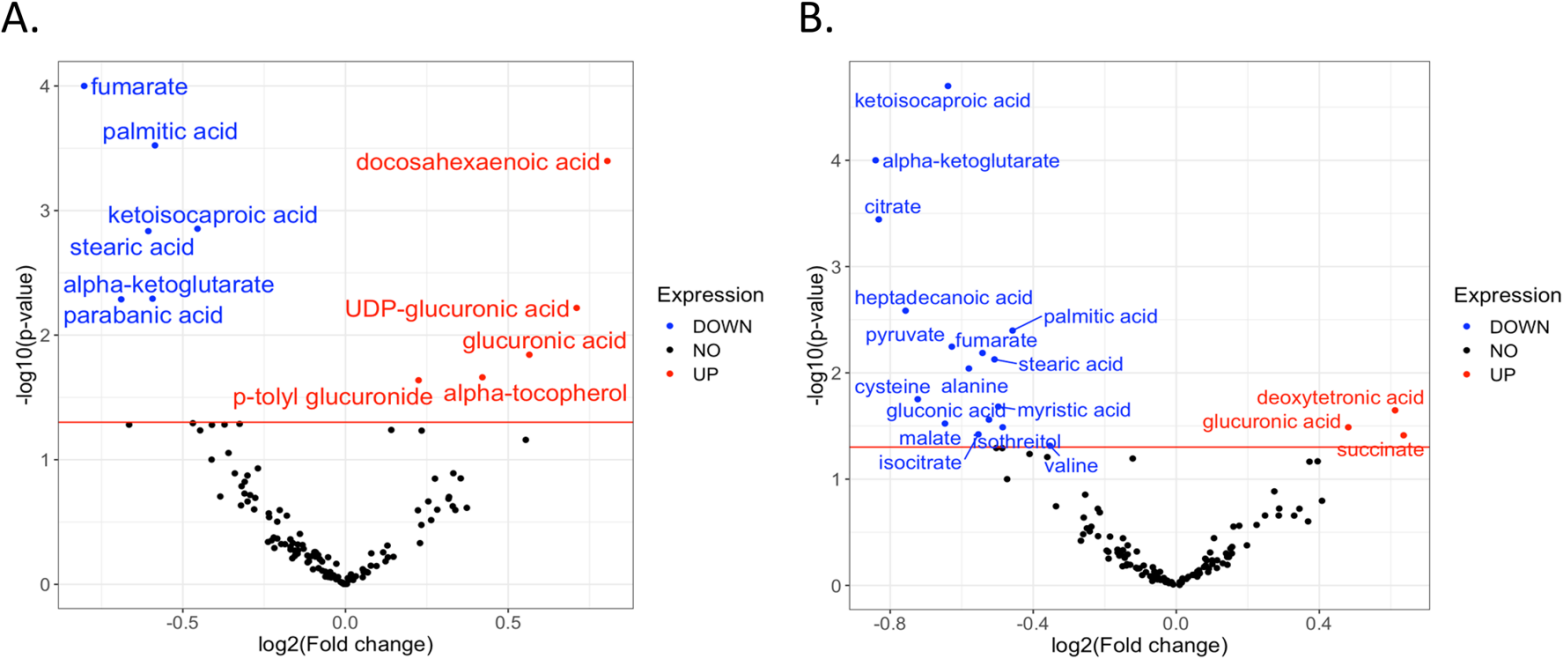
Volcano plots of changes in primary metabolites, relative to baseline, after EPA (**a**) and DHA (**b**) supplementation. Metabolites are shown in red if significantly increased following supplementation and in blue if significantly reduced following supplementation. Red line represents the cut-off point for FDR < 0.05.

**Figure 3:**
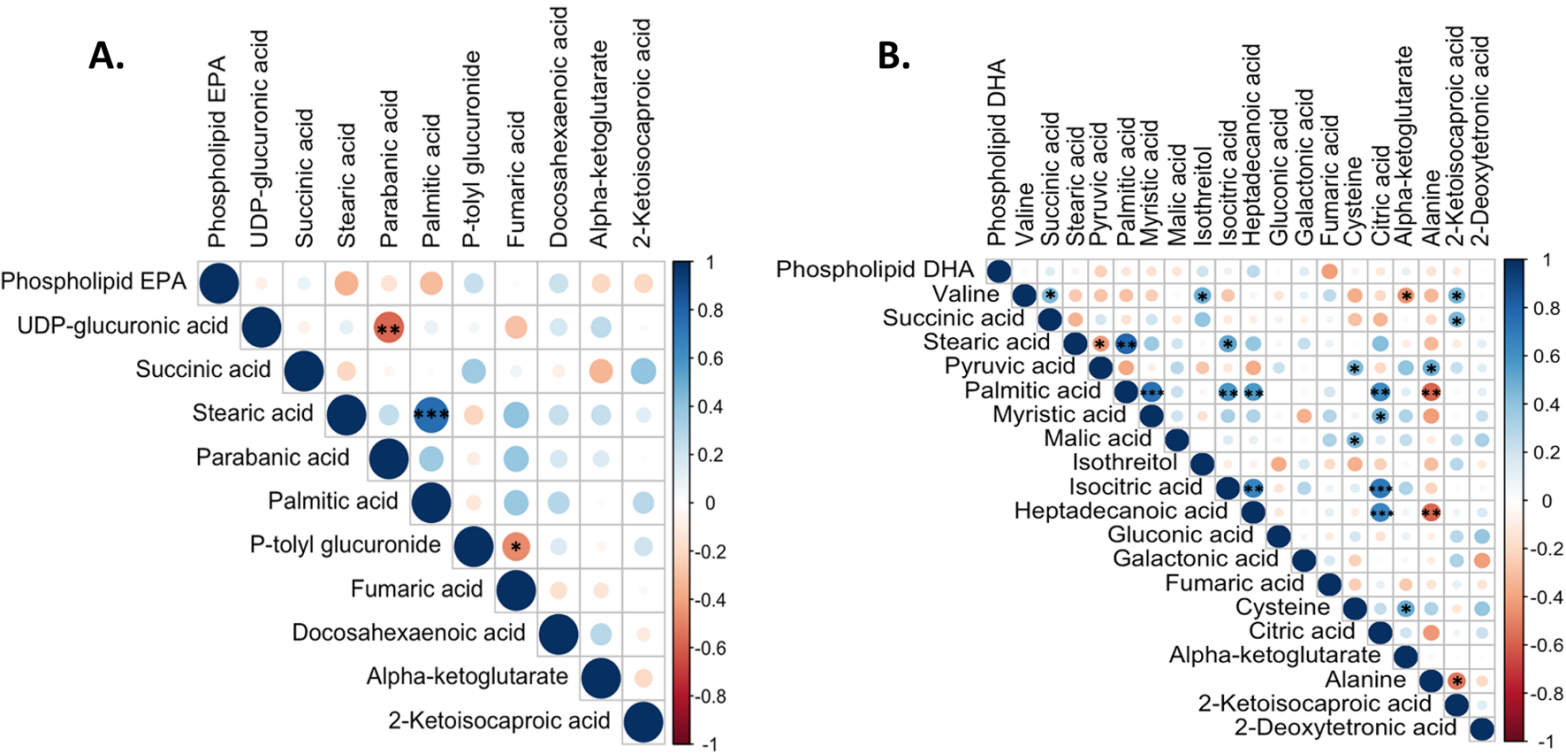
Correlation among metabolite changes after EPA and DHA supplementation. Metabolites shown to have significant changes after EPA and DHA supplementation were included for the Pearson’s correlation analysis. Positive and negative correlation are presented in blue and red, respectively. (**a**) Changes in metabolites and phospholipid EPA concentrations after EPA supplementation; (**b**) Changes in metabolites and phospholipid DHA concentrations after DHA supplementation. *P < 0.05, **P < 0.01, ***P < 0.001.

The PLS-DA plot of metabolites at baseline and after EPA and DHA supplementation is shown in Fig. 1. Relative to baseline, EPA supplementation significantly altered 11 known and 15 unknown serum primary metabolites (Table 3). EPA significantly increased non-esterified DHA and the glucose metabolites UDP-glucuronic acid and glucuronic acid. EPA lowered the tricarboxylic acid (TCA) cycle intermediate metabolites α-ketoglutarate and fumarate, the saturated fatty acids stearic acid and palmitic acid, and the leucine metabolite ketoisocaproic acid, in addition to parabanic acid (Table 3 and Fig. 2). The change in fumarate was inversely associated with the change in p-tolyl-glucuronide (Fig. 3).

**Table 3:** Peak height values of serum primary metabolites at baseline and after EPA and DHA supplementation (alphabetic order)^1^.

	Baseline	EPA	∆EPA from baseline^2^	DHA	∆DHA from baseline^2^	*FDR* ∆EPA vs. ∆DHA^3^
$\alpha$-Ketoglutarate	1572 (1374, 2098)	1344 (820, 1699)	− 546 (− 848, 156)**	1011 (814, 1303)	− 574 (− 1095, − 25)***	0.70
$\alpha$-Tocopherol	17,567 (15,450, 21,920)	21,166 (17,377, 24,927)	3599 (244, 4918)*	21,490 (15,673, 23,604)	3495 (− 430, 5599)	0.042
Citric acid	67,091 (58,034, 76,758)	63,059 (55,048, 74,021)	− 5585 (− 12,624, 8242)	60,541 (46,556, 65,524)	− 10,005 (− 21,349, − 845)***	0.05
Cysteine	30,308 (28,004, 40,007)	31,598 (25,512, 38,123)	− 1645 (− 6057, 10,161)	28,160 (25,635, 30,844)	− 2545 (− 11,847, 3008)*	0.47
Docosahexaenoic acid	1324 (1002, 1617)	1833 (1384, 2472)	493 (272, 1180)***	1611 (1214, 1840)	278 (− 32, 572)	0.51
Deoxytetronic acid	1323 (681, 1690)	1235 (635, 1666)	− 88 (− 504, 273)	1485 (1306, 1948)	274 (− 66, 835)*	0.0342
Fumaric acid^4^	5539 (3793, 6117)	4073 (3408, 5116)	− 871 (− 2260, − 14)***	4216 (3605, 5460)	− 104 (− 1516, 754)**	0.0042
Gluconic acid	1288 (1105, 1608)	1269 (1055, 1558)	161 (− 164, 247)	1217 (1042, 1649)	− 45 (− 119, 145)*	0.23
Glucuronic acid	1856 (1481, 2717)	2782 (2254, 3370)	654 (101, 1153)*	2697 (2145, 3088)	580 (− 49, 1371)*	0.56
Heptadecanoic acid	7790 (6494, 8727)	7017 (6143, 8826)	− 560 (− 2055, 458)	6604 (5490, 7788)	− 697 (− 2400, 344)**	0.19
Isocitric aicd	1094 (1001, 1341)	1146 (948, 1281)	− 94 (− 241, 138)	979 (808, 1117)	− 152 (− 390, 24)*	0.23
Isothreitol	2072 (1580, 2886)	2418 (1580, 2972)	85 (− 318, 735)	1654 (1088, 2221)	− 330 (− 1013, 101)*	0.41
Ketoisocaproic acid	50,177 (42,537, 57,751)	48,120 (36,122, 50,704)	− 6050 (− 10,576, − 1164)**	41,822 (33,509, 50,332)	− 5555 (− 16,107, 100)***	0.92
Malic acid	1350 (1233, 1731)	1598 (1261, 1852)	− 94 (− 352, 427)	1386 (1040, 1600)	− 253 (− 444, 111)*	0.89
Myristic acid	7063 (5741, 7953)	6388 (4678, 7191)	− 769 (− 1620, 583)	6069 (5771, 8255)	− 798 (− 2198, 1192)*	0.73
P-tolyl glucuronide	232 (164, 364)	294 (197, 569)	67 (− 64, 350)*	248 (187, 408)	19 (− 64, 120)	0.0381
Palmitic acid	143,747 (130,251, 170,438)	134,008 (103,508, 143,473)	− 20,098 (− 31,503, − 4319)***	135,581 (118,709, 161,108)	− 13,121 (− 29,561, 12,833)**	0.13
Parabanic acid	4087 (2895, 4648)	3369 (2586, 4401)	− 718 (− 1379, − 6)**	3576 (2957, 4704)	322 (− 849, 845)	0.11
Phenylacetic acid	803 (612, 1223)	874 (671, 996)	− 29 (− 205, 348)	973 (762, 1241)	190 (9, 464)	0.0276
Phenylethylamine	374 (294, 513)	586 (317, 724)	130 (− 117, 379)	358 (221, 549)	− 22 (− 152, 76)	0.0011
Pyruvic acid	39,697 (20,995, 49,411)	27,746 (17,769, 39,011)	− 9691 (− 21,665, − 363)	14,831 (9284, 25,225)	− 15,509 (− 24,309, 1969)**	0.83
Serotonin	1845 (1009, 2652)	1864 (994, 2670)	− 96 (− 616, 678)	2204 (1543, 3054)	− 158 (− 341, 739)	0.99
Stearic acid	448,176 (414,026, 561,336)	460,347 (352,958, 512,616)	− 48,364 (− 97,126, − 6545)**	430,408 (409,245, 499,584)	474 (− 124,502, 51,301)**	0.86
Succinic acid	3303 (2833, 3744)	3440 (3037, 3823)	505 (− 233, 1029)	3560 (3328, 4422)	579 (3, 1716)*	0.93
UDP-glucuronic acid	797 (611, 1103)	1111 (935, 1578)	389 (14, 587)**	1107 (576, 1382)	320 (− 286, 643)	0.54
Valine	1,248,470 (1,134,926, 1,308,718)	1,234,454 (1,088,759, 1,356,740)	2072 (− 90,478, 102,381)	1,134,770 (1,095,117, 1,297,538)	− 11,483 (− 151,936, 71,861)*	0.68

^1^Values are unadjusted and reported as median (25th, 75th). To correct for skewed distribution, generalized log-transformation and auto-scaling were applied before analysis.

^2^Comparison of changes from baseline to EPA or DHA was assessed using a liner mixed-effects model. FDR-adjusted *P* value: * < 0.05, ** < 0.01, *** < 0.001.

^3^Pairwise comparison between EPA and DHA (changes form baseline) were conducted using the *lsmeansLT* function from the *lmerTest* package for the linear mixed effect model.

^4^Fumaric acid showed significant sequence effect (*p* = 0.0073). When there was a sequence effect, data from the first period is extracted to run ANCOVA analysis, with baseline value and age as covariates. EPA supplementation reduced level of fumaric acid (*p* = 0.0107) when sequence effect was taken into account.

Relative to baseline, DHA significantly affected 19 known and 22 unknown metabolites. DHA increased glucuronic acid, the TCA cycle metabolite succinic acid, and deoxytetronic acid and reduced the amino acids alanine, valine and cysteine, the TCA intermediate metabolites pyruvate, α-ketoglutarate, citric acid, isocitrate, fumarate and malate, and the saturated fatty acids myristic acid, palmitic acid and stearic acid, in addition to phenylethylamine (Table 3 and Fig. 2). During DHA supplementation, the reduction in citrate was significantly associated with the reduction in myristic, palmitic and heptadecanoic acids and isocitric acid (Fig. 3). Non-esterified DHA concentrations did not change significantly after DHA supplementation (FDR = 0.07).

DHA supplementation, relative to EPA, led to a more pronounced reduction in citric acid (− 15% vs. − 8%, respectively; FDR = 0.05) and greater increases in phenylacetic acid (+ 21% vs. + 9%, FDR = 0.027) and deoxytetronic acid (+ 22% vs. − 6%, FDR = 0.034). On the other hand, EPA had more prominent effects than DHA in decreasing fumarate (− 16% vs. − 2%, respectively; FDR = 0.004) and increasing p-tolyl β-D-glucuronide (+ 27% vs. + 7%, FDR = 0.038) and phenylethylamine (+ 56% vs. -5%, FDR = 0.002).

To gain insight into the unique pathways associated with the changes in primary metabolites induced by EPA and DHA supplementation, pathway analysis was performed. Compared with baseline, EPA supplementation significantly affected six pathways including: ascorbate and aldarate metabolism; pentose and glucuronate interconversions; TCA cycle; alanine, aspartate and glutamate metabolism; tyrosine metabolism; and amino sugar and nucleotide sugar metabolism (Table 4). DHA supplementation was significantly associated with four pathways, including: ascorbate and aldarate metabolism; TCA cycle; pentose and glucuronate interconversions; and alanine, aspartate and glutamate metabolism (Table 4).

**Table 4:** Top pathways affected by EPA and DHA supplementation.

	Total cmpd	Hits N	Hits metabolites	Direction	FDR	Impact
**EPA, relative to baseline**						
Ascorbate and aldarate metabolism	8	2	Glucuronate	(+)	0.01	1
			UDP-glucuronate	(+)		
Pentose and glucuronate interconversions	18	2	Glucuronate	(+)	0.01	0.25
			UDP-glucuronate	(+)		
Citrate cycle (TCA cycle)	20	2	α-Ketoglutarate	(−)	0.03	0.08
			Fumarate	(−)		
Alanine, aspartate and glutamate metabolism	28	2	α-Ketoglutarate	(−)	0.03	0.05
			Fumarate	(−)		
Tyrosine metabolism	42	1	Fumarate	(−)	0.023	0.02
Amino sugar and nucleotide sugar metabolism	37	1	UDP-glucuronate	(+)	0.023	0.02
**DHA, relative to baseline**						
Ascorbate and aldarate metabolism	8	1	Glucuronate	(+)	0.042	0.50
Citrate cycle (TCA cycle)	20	7	Pyruvate	(−)	0.042	0.30
			Citrate	(−)		
			Isocitrate	(−)		
			α-Ketoglutarate	(−)		
			Succinate	(+)		
			Fumarate	(−)		
			Malate	(−)		
Pentose and glucuronate interconversions	18	1	Glucuronate	(+)	0.042	0.10
Alanine, aspartate and glutamate metabolism	28	6	Alanine	(−)	0.042	0.05
			Pyruvate	(−)		
			Citrate	(−)		
			α-Ketoglutarate	(−)		
			Succinate	(+)		
			Fumarate	(−)		
**EPA vs. DHA**						
Citrate cycle (TCA cycle)	20	2	Citrate	(+)	0.018	0.12
			Fumarate	(−)		
Glyoxylate and dicarboxylate metabolism	32	1	Citrate	(+)	0.018	0.03
Alanine, aspartate and glutamate metabolism	28	2	Citrate	(+)	0.018	0.002
			Fumarate	(−)		

Significant pathways (FDR < 0.05) with impact scores greater than 0 are presented.

**Figure 4:**
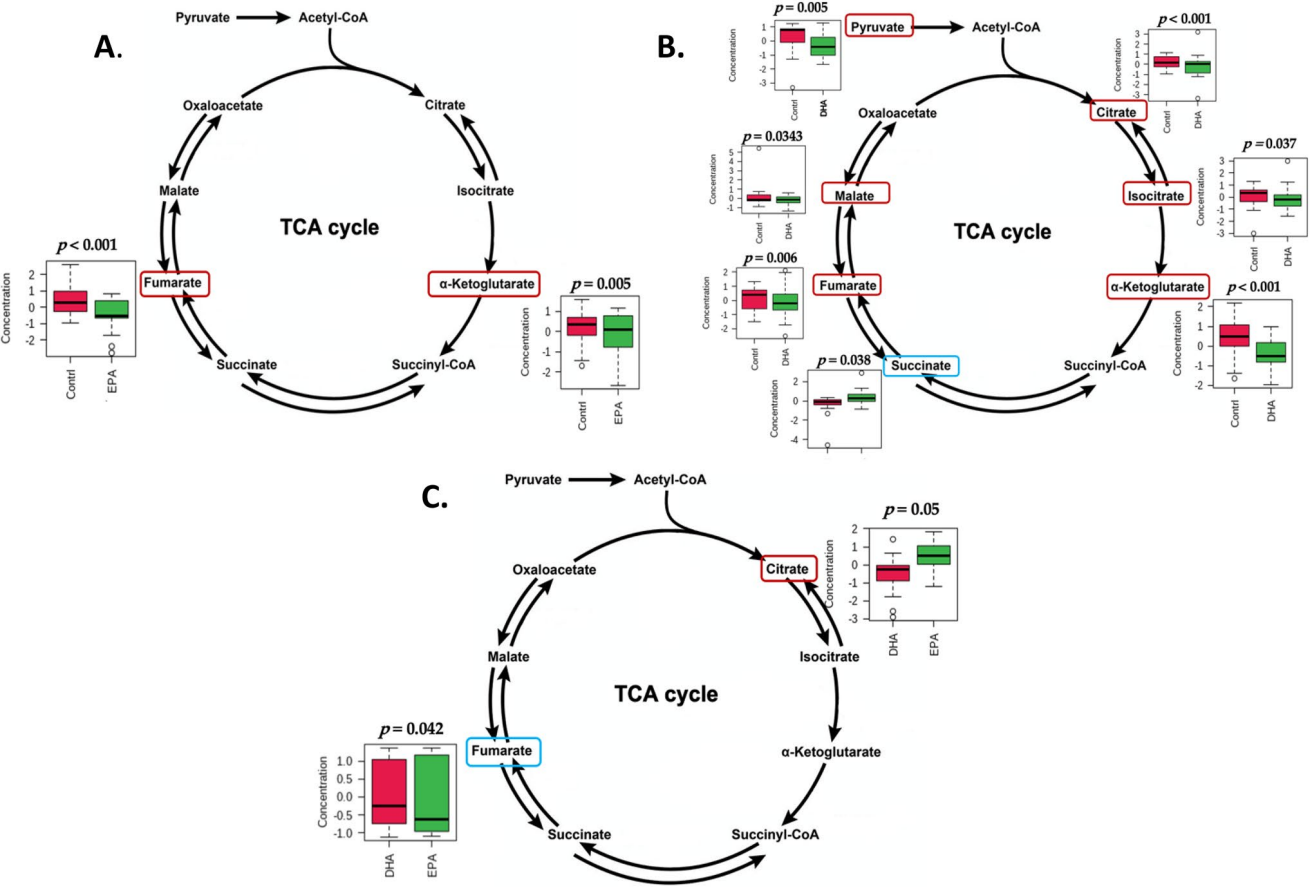
EPA and DHA modulation of TCA cycle metabolites. Significantly increased and decreased metabolites are circled in blue and red, respectively. (**a**) baseline versus EPA supplementation; (**b**) baseline versus DHA supplementation; (**c**) EPA versus DHA.

The TCA cycle was differentially affected by EPA and DHA supplementation, with a greater reduction of citrate by DHA and of fumarate by EPA (Table 4, Fig. 4).

### Effects of EPA and DHA on biogenic amines

Biogenic amine metabolites were measured in a subset of 10 subjects, whose characteristics are shown in Supplementary Tables 1 and 2. Compared to baseline, EPA supplementation significantly changed 255 metabolites (10 known and 245 unknown) (Supplementary Table 3). EPA significantly increased benzophenone and aminomethyl-cyclohexane-carboxylic acid and lowered alanine, creatinine, diamino-2-methylpropane, glutamate-glutamine, histamine, pyroglutamic acid, and stachydrine. DHA supplementation significantly affected a distinct set of metabolites (15 known and 343 unknown). DHA lowered acetyl-carnitine, L-propionylcarnitine, hydroxy-isovaleroylcarnitine, dihydrouracil, guanine, homoarginine, methylproline, methylumbelliferone, pyrazolyl-alanine, stachydrine, and triethanolamine, and increased serine, glycine and octanoylcarnitine.

DHA, relative to EPA, raised glycine (+ 29% vs. + 13%, respectively; FDR < 0.002) and serine (+ 12% vs. − 0.1%, FDR = 0.003), and reduced dihydrouracil (− 45% vs. − 20%, FDR < 0.0005) and stachydrine (− 31% vs. − 14%, FDR < 0.0001) to a greater extent than EPA.

There was no significant pathway associated with the changes in biogenic amines during EPA and DHA supplementation.

## Discussion

At baseline, participating subjects had low-grade inflammation and some characteristics of metabolic syndrome. They presented with high AA and low EPA and DHA in plasma phospholipids, in agreement with their inflammatory status^12^. Supplementation with both EPA and DHA led to significant changes in the concentrations of several serum metabolites that are tightly associated with various biological and metabolic pathways, with DHA affecting a greater number of metabolites than EPA.

In the context of no significant change in total serum non-esterified FA (NEFA) by EPA and DHA, both n-3 FA lowered the non-esterified saturated myristic, palmitic and stearic acids but only EPA supplementation significantly increased non-esterified DHA. In the fasting state, serum NEFA are mostly derived from adipocyte TG lipolysis^13^. In TG, DHA is preferentially found at the sn-2 position, while saturated fatty acids can be found randomly at any position. Adipocyte TG lipase (ATGL), which catalyzes the first step of adipocyte lipolysis, preferentially cleaves fatty acids at the sn-2 position of TG^14^ and its activity has been shown to be regulated by n-3 FA^15^. The increase in serum non-esterified DHA observed after EPA supplementation, but not after DHA supplementation, may therefore signal a greater activation of ATGL by EPA. Alternatively, other enzymes such as phospholipase may also contribute to this effect.

N-3 FA are activators of peroxisome proliferator-activated receptor γ (PPARγ), a transcriptional factor up-regulating key genes involved in adipocyte lipogenesis and TG storage^16^. The reduction in non-esterified saturated FA after EPA and DHA supplementation, even in the context of no significant change in total NEFA, may in part be explained by adipocyte PPARy activation^17,18^. In addition, since after DHA supplementation the reduction in myristic, palmitic and heptadecanoic acids was associated with the reduction in the TCA metabolite citrate, improved metabolic health due to greater mitochondrial efficiency and adipose tissue insulin sensitivity may explain our findings.

The TCA cycle, operating in the mitochondrial matrix, plays a critical role in the integration of several anabolic and catabolic pathways^19^. It serves as the primary source for the production of ATP, mostly through its linkage to oxidative phosphorylation^20,21^. In mice, excessive caloric intake or feeding a high-fat diet was associated with elevated TCA cycle activity, paralleled by mitochondrial dysfunction and increased oxidative stress and inflammation^22^. Our data show that 10-week supplementation with both EPA and DHA resulted in the reduction of two key TCA cycle intermediates, $\alpha$-ketoglutarate and fumarate, and that DHA supplementation led to a further significant reduction of four TCA cycle intermediates, including pyruvate, citrate, isocitrate and malate. Considering that our subjects were overweight or obese and displayed several characteristics of metabolic syndrome, the reduction in TCA metabolites during n-3 FA supplementation may indicate a greater efficiency of the TCA cycle, possibly due to improvements in mitochondrial function or insulin sensitivity. DHA is incorporated into the inner and outer mitochondrial membranes and thus may affect mitochondrial membrane organization and functions, including membrane potential, respiration and reactive oxygen species (ROS) production^23^. DHA also decreases membrane viscosity and potentially alter lipid-lipid and lipid-protein interactions^23,24^. The effect of DHA on membrane viscosity is greater than that of EPA^25^. Furthermore, as PPAR activators, EPA and DHA may regulate mitochondrial function through regulation of mitochondrial gene expression: PPARγ activation increases mitochondrial DNA copy number, mitochondrial biogenesis and the expression of genes in the fatty acid β-oxidation pathway^26^. The greater reduction in citrate by DHA than EPA is in accordance with a previous animal study which showed that DHA supplementation, rather than EPA, significantly lowered hepatic citrate compared to a control Western diet^27^.

In contrast to the other TCA cycle metabolites, succinate was increased after DHA, but not EPA, supplementation. Recent evidence indicates that succinate is not only a metabolite in the TCA cycle but also a gut microbiota metabolite^28^. Succinate is involved in multiple pathways and has been linked to both health and a range of pathologic conditions^28^. Through binding to a plasma membrane G-protein coupled receptor on macrophages^29^, succinate has been shown to initiate the process of active resolution of acute inflammation in the context of obesity^30^. Whether the upregulation of succinate by DHA is caused by succinate-producing bacteria or changes in the host metabolism is not known.

New roles of TCA cycle intermediates in cellular signaling and activation of immune cells have been documented^31,32^. Acetyl-CoA, isocitrate and citrate can alter both innate and adaptive immune responses and stem cell function, and α-ketoglutarate was shown to repress pro-inflammatory responses while improving the anti-inflammatory profile in macrophages^32^. In contrast, succinate and fumarate may work as oncometabolites that promote tumorigenesis through the suppression of histone and DNA demethylation^32^. Given that our data show significant changes in these metabolites, more studies are needed to further elucidate their systemic effects on non-metabolic signaling and their association with diseases.

Glucuronate was significantly affected by EPA and DHA supplementation in relation to both the ascorbate and aldarate metabolism and the pentose and glucuronate interconversion pathway. EPA demonstrated a higher impact by increasing both glucuronate and UDP-glucuronate, while DHA only raised glucuronate. These metabolites are generated from glucose through a series of enzymatic reactions^33^. Conjugation with glucuronic acid is the most common phase-II reaction, with UDP-glucuronosyltransferases catalyzing the formation of glucuronides from a large variety of xenobiotics and endogenous substrates like bilirubin, making them more hydrophilic and easily excreted by the kidneys^34^. At low doses, DHA has been shown to reduce the activity of the UDP-glucuronosyltransferase A1 in newborn mice, leading to increased blood bilirubin levels^35^. At higher doses, however, DHA rather activated the enzyme, possibly through PPAR induction^35^. In our study, bilirubin levels were not affected by either DHA or EPA supplementation. Therefore, the increase in UDP-glucuronate and glucuronate levels by n-3 FA may be due to increased synthesis from glucose.

In the subset of 10 participants who had serum biogenic amines assessed, several metabolites were differently affected by EPA and DHA supplementation. EPA significantly reduced alanine levels, possibly due to reduced muscle protein breakdown. Creatinine levels, a marker of kidney function, was also reduced, but reductions in serum creatinine may also indicate reduced protein breakdown.

DHA supplementation changed the concentration of a greater number of metabolites than EPA. Specifically, DHA reduced plasma levels of short-chain acylcarnitines, including acetylcarnitine, propionylcarnitine and hydroxyisovalerylcarnitine. Patients with insulin resistance and type 2 diabetes tend to present with elevated levels of these acylcarnitines, which are considered a marker of inefficient β-oxidation and of mitochondrial dysfunction^36^. Reduction in acetylcarnitine following n-3 FA supplementation has been previously reported^37^. Studies have demonstrated that acetyl-CoA accumulates in conditions of TCA cycle overwork, leading to increased formation of acetylcarnitine^38^. In addition, propionylcarnitine and hydroxyisovalerylcarnitine are metabolites of amino acid metabolism and their reduction may indicate reduced protein breakdown. Taken together, our findings suggest improved β-oxidation, and mitochondrial function, and reduced protein breakdown by DHA. On the other hand, the finding of increased octanoylcreatinine by DHA remains unexplained in the context of the reduction of the other acylcarnitines.

DHA supplementation was also associated with increased levels of glycine and serine. In the serine synthesis pathway, glucose is converted to 3P-pyruvate, and, with glutamate, 3P-pyruvate is converted to serine and then glycine^39^. In endothelial cells, this pathway leads to the synthesis of heme in the mitochondria and results in improved mitochondrial function and reduced oxidative stress^39^.

In conclusion, we observed significant and shared effects of EPA and DHA on metabolites related to lipid and adipocyte metabolism, TCA cycle efficiency, mitochondrial function and detoxification processes in subjects with chronic inflammation and some key aspects of metabolic syndrome. However, EPA and DHA also exhibited differential effects on the serum metabolome as evidenced by a greater number of metabolites affected by DHA than EPA and a significantly greater effect on selected metabolites by DHA or EPA.

Strengths of our study include the randomized, controlled, crossover study design, the high compliance of participants, and the assessment of the metabolic effects of EPA and DHA effects in subjects with chronic inflammation, a population at higher risk of cardiometabolic diseases. Limitations are the small sample size and the lack of a healthy control group.

## Methods

### Study design

The details of the study design and related primary outcomes have been published previously^11^. The study had a randomized, double-blind, controlled, crossover design and consisted of an initial 4-week lead-in phase during which the subjects were given 3 g/day of high-oleic acid sunflower oil as a control supplement, followed by randomization to a 10-week supplementation with either EPA or DHA (3 g/day) and crossover after a 10-week washout phase. The random allocation sequence was generated by REDCap. Subjects, study investigators and laboratory personnel were blind to treatment. The end of the 4-week lead-in phase was defined as baseline. Ethyl ester forms of EPA and DHA were provided in capsules containing 750 mg/capsule with a purity > 97% (Prevention Pharmaceuticals Inc, CT). Each participant was instructed to take a total of four capsules daily, two with the morning meal, and two with the evening meal. Subjects’ compliance was assessed by the number of returned capsules at the end of each phase and was greater than 80% in all participants. All research was performed in accordance with the Declaration of Helsinki.

### Study subjects

Subjects were studied at the Jean Mayer USDA Human Nutrition Research Center on Aging at Tufts University. Between April 2016 and November 2017, 24 participants were enrolled and 21subjects (9 men and 12 postmenopausal women) completed the study. Three subjects dropped out of the study during the placebo lead-in phase. Subjects, aged 50–75 years and meeting the following inclusion criteria, completed the study: (1) serum C-reactive protein (CRP) concentrations $\ge$ 2 g$\mu$/mL; (2) fasting plasma TG concentrations between 90 and 500 mg/dL; and (3) at least one of the criteria for the modified definition of metabolic syndrome including abdominal obesity (waist circumference $\ge$ 102 cm for men and $\ge$ 89 cm for women), hypertension (blood pressure $\ge$ 130/80 mmHg or use of antihypertensive medications), and fasting blood glucose concentrations 100–125 mg/dL^11^. Subjects were excluded if they had consumed either fish more than 2 times/week or supplements containing fish oil or EPA/DHA during the 6 months prior to the trial; were allergic to sardines and/or sunflower oil; had regularly used anti-inflammatory medications such as NSAID or were under anticoagulant therapy; had other known diseases that might alter their metabolic status such as kidney or liver diseases; were alcohol users (> 7 drinks/week) or smokers. Subjects were instructed to follow a low-saturated fat diet (25–35% of calories from total fat, < 7% from saturated fat, and cholesterol < 200 mg/day) and keep their physical activity levels unchanged throughout the study. Subjects were also asked not to consume more than 2 fish meals/week and to abstain from taking fish oil supplements during the study. To enhance adherence to dietary and lifestyle instructions, consultations with a registered dietitian were carried out at baseline and at each study visit. The study protocol was approved by the Tufts Health Sciences Institutional Review Board and is registered at clinicialtrials.gov (NCT02670382, 01/02/2016). All study participants provided informed consent.

### Blood sample collection

Twelve-hour fasting venous blood samples were collected into silica-coated tubes (Becton Dickinson, NJ) at baseline and the end of each supplementation phase. After being kept at room temperature for 20 min to allow coagulation to occur, blood was centrifuged (1,000 g for 15 min at 4 °C) and serum was aliquoted and immediately stored at − 80 °C for further analysis.

All methods used in the current study for the assessment of biochemical parameters and metabolites have been previously described and have been carried out in accordance with relevant guidelines and regulations**.** The effects of EPA and DHA on the serum metabolome was intended as an exploratory analysis.

### Measurement of biochemical parameters

Serum concentrations of TG, glucose, albumin, and other blood chemistry analytes were assessed at the screening visit and at the end of each phase using commercial reagents on an AU480 Chemistry Analyzer (Beckman Coulter Inc, CA)^11^.

### Metabolic profiling

#### Serum primary metabolites

Primary metabolites, including a panel of carbohydrates and sugar phosphates, amino acids, hydroxyl acids, free fatty acids, purines, pyrimidines, aromatics, and exposome-derived chemicals, were measured in fasting serum samples, obtained at baseline and at the end of each supplementation phase from all 21 subjects, by gas chromatography-time of flight mass spectrometry (GC-TOF MS) by West Coast Metabolomics^40,41^. Briefly, serum samples were extracted and derivatized using acetonitrile:isopropanol:water (3∶3∶2; v/v/v), centrifuged and dried. A set of 13 C8–C30 fatty acid methyl ester internal standards were added, and samples were derivatized by 10 µL methoxyamine hydrochloride in pyridine followed by 90 µL MSTFA. Analytes were separated using an Agilent 6890 gas chromatograph (Santa Clara, CA) equipped with a 30 m long, 0.25 mm i.d. Rtx5Sil-MS column with 0.25 µm 5% diphenyl film and additional 10 m integrated guard column (Restek, Bellefonte PA). Mass spectrometry was performed by a Leco Pegasus IV time of flight mass spectrometer (St. Joseph, MI). Mass spectra were acquired from m/z 85–500 at 17 spectra s^−1^ and 1850 V detector voltage. Raw data files were exported and validated using the metabolomics BinBase database (BinBase.com, Ltd)^41^, combined with the validity of chromatogram (< 10 peaks with intensity > 10^7 counts s-1), detection of unbiased retention index marker (MS similarity > 800 with high m/z marker ions) and retention index calculation by 5^th^ order polynomial regression, as previously described^40^. This method identified 130 known and 276 unknown primary metabolites in all serum samples from all participants.

#### Serum biogenic amines

Serum biogenic amines were measured only in a subset of 10 randomly selected participants, due to budget restrictions. Biogenic amines, comprising acylcarnitines, TMAO, cholines, betaines, SAM, SAH, nucleotides and nucleosides and methylated and acetylated amines, were assessed by West Coast Metabolomics by HILIC-QTOF MS/MS, as previously described^42^. Hydrophilic interaction liquid chromatography (HILIC) method was used. Briefly, serum samples were extracted and derivatized. Five µL of re‐suspended samples were injected onto a Waters Acquity UPLC BEH Amide column (150 mm length × 2.1 mm id; 1.7 µm particle size) with an additional Waters Acquity VanGuard BEH Amide pre‐column (5 mm × 2.1 mm id; 1.7 µm particle size) coupled to an Agilent 1290 Infinity UHPLC. The mobile phases were prepared with 10 mm ammonium formate and 0.125% formic acid (Sigma‐Aldrich) in either 100% LC‐MS grade water for mobile phase (A) or acetonitrile: water (95:5, v/v) for mobile phase (B). Spectra were collected using Q Exactive HF mass spectrometer in positive ion mode. Quality control samples (Standard Reference Material NIST plasma samples) were included. For metabolite identification, HILIC spectral library of authentic standards was used in addition to *m/z* and retention time database. A total of 178 known and 2,946 unknown biogenic metabolites were measured.

## Data analysis

Results are presented as mean and standard deviation (SD) or median (25^th^ and 75^th^ percentile). Metabolite data were subjected to generalized log transformation and auto-scaling before statistical analyses due to skewness. Statistical analyses, including partial least square discriminant analysis (PLS-DA), were conducted in R version 4.0.0 (R core team, 2020). Comparisons of the change in serum metabolites from baseline to EPA versus DHA supplementation were evaluated using a linear mixed-effects model, by the *lmer* function from the *lme4* package. The model accounted for the random effect of subjects and the fixed effects of treatment, period, and sequence. Baseline values and age were included in the model as covariates. The effect of EPA or DHA supplementation versus baseline was assessed by using the *lsmeansLT* function from the *lmerTest* package, which allows for comparisons of least square means between baseline and each supplemental phase in the linear mixed model. When there was a significant sequence effect, data from only the first supplementation period was selected to perform ANCOVA for the comparisons between EPA and DHA and paired t-tests for the comparisons between baseline versus each treatment, with the inclusion of baseline concentration as a covariate. No sex differences were observed in the changes of metabolites following EPA and DHA supplementation, therefore men and women were combined in our analyses. To correct for multiple testing, false discovery rate (FDR) was used to determine adjusted *p*-values. Statistical significance was defined as FDR < 0.05.

MetaboAnalyst 4.0 (https://www.metaboanalyst.ca), a web-based platform for comprehensive analysis of quantitative metabolomic data, was used to perform pathway analysis and visualize the biological pathways associated with significantly affected metabolites. The pathway analysis algorithms are based on the hypergeometric test for over-representation analysis, and relative-betweenness centrality for pathway topology analysis. Hypergeometric test compares the number of significant metabolites within a specific pathway with the expected value^43^. Results included the impact value of the metabolic pathway based on the Kyoto Encyclopedia of Genes and Genomes (KEGG) for Homo sapiens database that were most relevant to the selected metabolites^44^. Pathways with an impact score > 0 and FDR $<$ 0.05 were considered statistically significant.

To identify metabolites with similar or opposite changes after EPA and DHA supplementation, Pearson’s correlation coefficient analysis was conducted. Significantly changed metabolites from the linear-mixed model were chosen and the coefficients were calculated across metabolite pairs. In addition, the change in concentration of each metabolite was correlated with the change in plasma phospholipid EPA or DHA mol percentage. The correlation coefficients (*r*) of metabolites within each feature, which reflect the strength of their relationships, were ranked from high to low using the pattern searching function. Heatmaps were created to provide an overview and visualization of the correlation matrix. An absolute value of *r* that is greater than 0.6 indicated a moderate to strong linear relationship, and *P* < 0.05 was considered statically significant.

## Supplementary Information

Supplementary Information.
