# Supplementary material for: Differential and shared effects of eicosapentaenoic acid and docosahexaenoic acid on serum metabolome in subjects with chronic inflammation
Source: Sci Rep. 2021 Aug 11;11:16324. doi: 10.1038/s41598-021-95590-7 (PMC8357808; doi:10.1038/s41598-021-95590-7)
Supplement: Supplementary file 1 — Supplementary Information. [file 41598_2021_95590_MOESM1_ESM.docx]

**Supplementary data**

**Differential and shared effects of eicosapentaenoic acid and docosahexaenoic acid on serum metabolome in subjects with chronic inflammation**

**Wan-Chi Chang, Jisun So, Stefania Lamon-Fava**

| **Supplementary table 1. Characteristics of subjects at screening visit (N=10)^1^** | | | |
| --- | --- | --- | --- |
|  | All (*n* = 10) | Male (*n* = 4) | Female (*n* = 6) |
| Age (y) | 64 ± 6 | 62 ± 5 | 65 ± 6 |
| Weight (kg) | 86.5 ± 7.9 | 98.4 ±18.1 | 78.5 ± 14.8 |
| BMI (kg/m^2^) | 29.1 ± 3.1 | 30.0 ± 3.7 | 28.5 ± 2.8 |
| Waist circumference (cm) | 99 ± 13 | 109 ± 10 | 93 ± 12 |
| SBP (mmHg) | 131 ± 21 | 126 ± 11 | 134 ± 25 |
| DBP (mmHg) | 82 ± 14 | 90 ± 10 | 76 ± 14 |
| Fasting glucose (mg/dL) | 99 ± 11 | 97 ± 12 | 101 ± 12 |
| Fasting TG (mg/dL) | 143 ± 41 | 123 ± 17 | 157 ± 48 |
| ^1^ Values are reported as mean ± SD. BMI, body mass index; SBP, systolic blood pressure; DBP, diastolic blood pressure; TG, triglycerides. | | | |

| **Supplementary table 2. Serum concentrations of biochemical parameters at baseline and after EPA and DHA supplementation (n=10)^1^** | | | | | | |
| --- | --- | --- | --- | --- | --- | --- |
|  | **Baseline** | **EPA** | **∆EPA from baseline^2^** | **DHA** | **∆DHA from baseline^2^** | ***P*-∆EPA vs. ∆DHA^3^** |
| Albumin (g/dL) | 4.10 ± 0.23 | 4.16 ± 0.17 | 0.05 ± 0.24 | 4.23 ± 0.14 | 0.12 ± 0.16 | 0.17 |
| Total protein (g/dL) | 6.63 ± 0.32 | 6.70 ± 0.36 | 0.07 ± 0.38 | 6.81 ± 0.23 | 0.18 ± 0.29 | 0.13 |
| SGPT (mU/mL) | 6.80 ± 2.39 | 8.60 ± 3.41 | 1.80 ± 3.33 | 8.90 ± 3.11 | 2.10 ± 1.79 | 0.46 |
| SGOT (mU/mL) | 13.40 ± 3.24 | 14.40 ± 4.20 | 1.00 ± 3.59 | 14.90 ± 3.28 | 1.50 ± 2.17 | 0.29 |
| LDH (U/L) | 111.70 ± 21.38 | 119.70 ± 13.73 | 8.00 ± 18.57 | 115.30 ± 17.94 | 3.60 ± 9.18 | 0.17 |
| ALP (U/L) | 55.10 ± 19.03 | 56.40 ± 21.04 | 1.30 ± 12.17 | 57.00 ± 23.30 | 1.90 ± 9.12 | 0.65 |
| BUN (mg/dL) | 16.90 ± 4.56 | 15.50 ± 3.10 | -1.40 ± 2.80 | 15.90 ± 4.15 | -1.00 ± 2.94 | 0.71 |
| Glucose (mg/dL) | 97.80 ± 9.00 | 98.50 ± 7.20 | 0.70 ± 6.99 | 98.50 ± 8.90 | 0.70 ± 3.83 | 0.98 |
| NEFA (mmol/L) | 0.15 ± 0.06 | 0.18 ± 0.11 | 0.02 ± 0.10 | 0.15 ± 0.09 | -0.01 ± 0.06 | 0.39 |
| Uric acid (mg/dL)^6^ | 6.17 ± 1.50 | 6.20 ± 1.54 | 0.03 ± 0.75 | 6.12 ± 1.44 | -0.05 ± 0.64 | 0.96 |
| Total bilirubin (mg/dL) | 0.69 ± 0.17 | 0.69 ± 0.14 | 0.10 ± 0.19 | 0.63 ± 0.14 | 0.04 ± 0.23 | 0.41 |
|  | | | | | | |
| ^1^ Values are reported as unadjusted mean ± SD. Log-transformation was applied before analysis. | | | | | | |
| ^2^ Comparisons of changes from baseline to post- EPA and DHA supplementation were evaluated using a linear mixed-effects model. FDR correction was applied. | | | | | | |
| ^3^ Pairwise comparisons between the changes from baseline to either EPA or DHA were conducted using the *lsmeansLT* function from the *lmerTest* package for the linear mixed effect model. | | | | | | |
| SGPT, serum glutamic-pyruvic transaminase; SGOT, serum glutamic-oxaloacetic transaminase; LDH, lactate dehydrogenase; ALP, alkaline phosphatase; BUN, blood urea nitrogen. | | | | | | |
|  | | | | | | |

| **Supplementary table 3. Peak height of serum biogenic amine metabolites at baseline and after EPA and DHA supplementation (n=10)^1,2^** | | | | | | | | | | | | |  |
| --- | --- | --- | --- | --- | --- | --- | --- | --- | --- | --- | --- | --- | --- |
|  | **Baseline** | | **EPA** | | **∆EPA from baseline ^2^** | | **DHA** | | **∆DHA from baseline ^2^** | | ***P*-∆EPA vs. ∆DHA^3^** | |  |
| Alanine | | 10581110 (7976974,11756231) | | 8733491 (7883291,9964650) | | -1853195 (-3298105,1095616)* | | 9443019 (7992521,10693786) | | -213698 (-2024336,1367703) | | 0.13 | |
| Acetylcarnitine | | 84986784 (73522962,92671740) | | 70697480 (58033330,85093912) | | -15832232 (-19206364,14084535) | | 59651666 (50799830,82139020) | | -19525992 (-39878409,1354778)* | | 0.68 | |
| Aminomethylcyclohexane-carboxylic acid | | 715464 (446004,894216) | | 1099382 (931596,1402218) | | 449088 (156219,870534)* | | 790337 (576173,916465) | | 101264 (-321521,412276) | | 0.45 | |
| Benzophenone | | 110587 (84603,150720) | | 176912 (102273,244174) | | 106573 (38762,149139)** | | 121992 (86413,191471) | | -29287 (-85026,67225) | | 0.015 | |
| Bifonazole | | 25413 (6310,40168) | | 337 (337,5303) | | -8686 (-25756,-3682)* | | 7679 (337,76422) | | -1982 (-41630,39122) | | 0.51 | |
| Creatinine ^4^ | | 333559264 (291725416,403745160) | | 314416576 (291048544,401424656) | | -39215120 (2132824,97550648)* | | 323593088 (308097352,411090480) | | -10670432 (-96264816,65531744) | | 0.88 | |
| Diamino-2-methylpropane | | 1057411 (914950,1189572) | | 981971 (827097,1103154) | | -85548 (-210584,64649)* | | 969200 (864199,1150495) | | 29093 (-291890,130543) | | 0.0013 | |
| Dihydrouracil | | 24816 (15921,39617) | | 19364 (14133,24910) | | 1005 (-10032,10524) | | 13203 (9144,32332) | | -17168 (-35182,9085)** | | 0.0005 | |
| Glu.Gln | | 221476 (208134,273834) | | 220963 (172156,270812) | | -3259 (-58717,21927)* | | 257660 (245516,295931) | | 41367 (-65003,102206) | | 0.0213 | |
| Glycine | | 1240624 (1148567,1549835) | | 1413124 (1098587,1581916) | | -22289 (-416606,304667) | | 1592622 (1422419,1738478) | | 453062 (297354,732733)** | | 0.0016 | |
| Guanine ^4^ | | 59694 (43991,138439) | | 43120 (34734,76726) | | -3656 (-41018,40576) | | 54495 (18591,109820) | | -35179 (-134331,12518)* | | 0.41 | |
| Histamine | | 53616 (22701,73085) | | 34240 (29745,45040) | | -18885 (-133689,10903)* | | 35779 (29354,49958) | | -1158 (-31316,17302) | | 0.11 | |
| Homoarginine | | 1872491 (1312860,2614747) | | 1752544 (1445664,2111588) | | 163275 (-113746,569572) | | 1646289 (1472402,1928662) | | -1035172 (-1427974,-378824)** | | 0.01 | |
| Hydroxyisovaleroyl-carnitine | | 152875 (82984,205032) | | 89481 (82340,177651) | | 12398 (-37787,39578) | | 76625 (65393,177855) | | -73108 (-151450,-9598)* | | 0.04 | |
| Lysine | | 18756444 (15651783,19012859) | | 18471334 (16372627,20528291) | | 152261 (-2995813,3418278) | | 18260290 (16595663,23278533) | | 241993 (-1552746,3958847) | | 0.007 | |
| Lisdexamfetamine | | 286037 (250894,324977) | | 293812 (263432,321933) | | 3488 (-45222,59476) | | 304265 (263200,360609) | | 12543 (-24951,61385) | | 0.008 | |
| L-Propionylcarnitine | | 6187159 (5468882,6826572) | | 5641327 (4535310,6975147) | | 402296 (-2043255,1212585) | | 4821018 (3651778,5551820) | | -1630429 (-2565920,-473915)* | | 0.45 | |
| Nandrolone | | 11295 (390,30313) | | 10809 (1450,39829) | | -31439 (-79935,-573) | | 19818 (7606,33992) | | 4959 (33,33602)* | | 0.03 | |
| Methylproline | | 1013022 (886785,3121997) | | 1133332 (860004,2236308) | | 451810 (72807,1329996) | | 943645 (821099,3044799) | | -2272983 (-4497700,-263584)*** | | 0.003 | |
| Methylumbelliferone ^4^ | | 15890 (11137,22519) | | 9496 (6428,11287) | | -3899 (-8464,727) | | 7576 (4224,11221) | | -14733 (-21247,-3702)*** | | 0.04 | |
| Octanoylcarnitine | | 562370 (383447,675775) | | 445630 (286590,618515) | | 97566 (-225724,149722) | | 762583 (571776,1240967) | | 497663 (-83549,706976)* | | 0.02 | |
| Ornithine | | 5413993 (4801550,6599121) | | 4969386 (4600790,6158913) | | -362918 (-2099503,1204443) | | 5234870 (5264263,6921657) | | -242095 (-89354,1814587) | | 0.003 | |
| Pyroglutamic acid | | 2097568 (1777534,2516711) | | 2021384 (1759870,2562910) | | -295987 (-554283,179361)* | | 2063661 (1853228,2335474) | | 188217 (47547,464623) | | 0.002 | |
| Pyrazolyl alanine | | 3628 (1920,87243) | | 13828 (3348,37783) | | 10967 (1117,35280) | | 1305 (550,38965) | | -17219 (-109775,-3461)* | | 0.002 | |
| Serine | | 1647232 (1574627,1743869) | | 1670660 (1509970,1779988) | | -2180 (-165801,23568) | | 1887844 (1722826,1984306) | | 193771 (99828,418773)** | | 0.001 | |
| Stachydrine | | 21613227 (8413727,97821784) | | 18523995 (6190437,84737578) | | -7984145 (-81086553,2287385)* | | 14806191 (6339343,81805564) | | -8879296 (-93247623,-24692634)** | | 0.0001 | |
| Triethanolamine | | 1296125 (1139156,1969328) | | 1107504 (678588,1522139) | | -105682 (-547901,202880) | | 1072099 (562199,1464799) | | -833350 (-2931256,-616156)*** | | 0.06 | |
| ^1^ Values are unadjusted and reported as median (25^th^, 75^th^).To correct for skewed distribution, generalized log transformation and auto-scaling were applied before analyses. | | | | | | | | | | | | |  |
| ^2^ Comparisons of serum metabolite changes from baseline to post- EPA and DHA were evaluated using a linear mixed-effects model. FDR-adjusted P value: * < 0.05, ** < 0.01, *** < 0.001. | | | | | | | | | | | | |  |
| ^3^ Pairwise comparisons between the changes from baseline to either EPA or DHA were conducted using the *lsmeansLT* function from the *lmerTest* package for the linear mixed effect model. | | | | | | | | | | | | |  |

| ^4^ Methylumbelliferone showed significant age effect (p = 0.0132). Creatinine (p = 0.0006) and Guanine (p = 0.0122) showed significant sequence effect. When there was a sequence effect, data from period 1 was extracted to run ANCOVA analysis, with baseline value and age as covariates. Treatment had a significant effect on creatinine acid (p = 0.0117) but not on guanine (p = 0.19). |
| --- |
